# A Case Report: Idiopathic or Drug-Induced Autoimmune Hepatitis—Can We Draw a Line?

**DOI:** 10.3390/clinpract13060125

**Published:** 2023-11-13

**Authors:** Dorotea Božić, Ante Tonkić, Katarina Vukojevic, Maja Radman

**Affiliations:** 1 Department of Gastroenterology, University Hospital of Split, Spinčićeva 1, 21000 Split, Croatia; dbozic@kbsplit.hr; 2 Department of Endocrinology, University Hospital of Split, Spinčićeva 1, 21000 Split, Croatia; ante.tonkic1@mefst.hr (A.T.J.); maja.radman1@st.htnet.hr (M.R.); 3 Department of Anatomy, Histology and Embryology, University of Split School of Medicine, 21000 Split, Croatia

**Keywords:** drug-induced autoimmune hepatitis, AIH, DILI, acute hepatitis

## Abstract

Idiosyncratic drug-induced liver injury (DILI) is an unpredictable reaction of individuals exposed to a certain drug, and drug-induced autoimmune hepatitis (DIAIH) presents a DILI phenotype that mimics idiopathic autoimmune hepatitis (AIH) when considering the clinical, biochemical, serological and histological parameters. We present a case report of a 48-year-old male who was hospitalized due to severe hepatocellular liver injury two months after self-treatment with a muscle-building dietary supplement based on arginine-alpha-ketoglutarate, L-citrulline, L tyrosine, creatine malate and beet extract. His immunology panel was positive with increased IgG levels, and radiologic methods showed no signs of chronic liver disease. He underwent corticosteroid treatment with adequate response. After therapy withdrawal, a clinical relapse occurred. Seven months after the initial presentation, liver MR suggested initial cirrhotic changes in the right liver lobe. A liver biopsy revealed abundant lymphoplasmacytic infiltrate with piecemeal necrosis and grade 2 fibrosis. He responded well to the corticosteroid treatment again, and was further treated with low-dose prednisone without additional relapses. Several years later, further management confirmed the presence of liver cirrhosis with no histological or biochemical signs of disease activity. DIAIH is a DILI phenotype that is difficult to distinguish from idiopathic AIH despite a wide armamentarium of diagnostic methods. It should always be considered among the differential diagnoses in patients presenting with hepatocellular liver injury.

## 1. Introduction

Idiosyncratic drug-induced liver injury (DILI) is an unpredictable reaction of individuals exposed to a certain drug, with a variable latency and wide spectrum of clinical and pathohistological presentations, ranging from mild asymptomatic liver injury to acute liver failure requiring transplantation [1]. According to the results of the Spanish DILI Registry from 2021, herbal and dietary products are responsibile for approximately 3.4% of DILI cases. According to the US Drug-Induced Liver Injury Network (DILIN) and the Iceland study, these rates were significantly higher in previous decades, when they reached 16% [2,3,4].

Drug-induced autoimmune hepatitis (DIAIH) presents a DILI phenotype that mimics idiopathic autoimmune hepatitis when considering the clinical, biochemical, serological and histological parameters [1]. Herein, we present a case of a 48-year-old male who was hospitalized due to severe hepatocellular liver injury two months after self-treatment with a muscle-building dietary supplement. 

**Figure 1:**
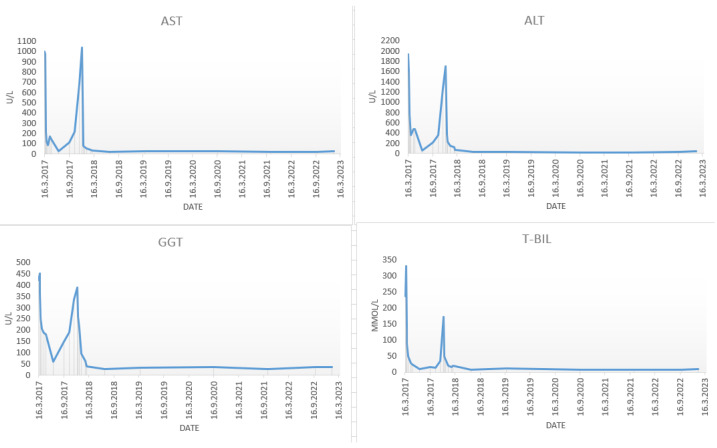
Trend of laboratory findings. Dynamics of liver enzymes (AST, ALT, GGT) and bilirubin levels from the initial admission until the present time. AST: aspartate aminotransferase; ALT: alanine aminotransferase; GGT: gamma glutamyl transferase; T-BIL: total bilirubin.

In March 2017, a 48-year-old male was admitted to the department of gastroenterology and hepatology due to severe liver injury. He had no previous liver disease or other chronic diseases. The patient reported recently taking a dietary supplement for muscle mass growth based on arginine-alpha-ketoglutarate, L-citrulline, L-tyrosine, creatine malate and beet extract. Upon admission, he presented with malaise and icterus. Laboratory parameters revealed increased bilirubin and aminotransferase levels, with normal alkaline phosphatase (Figure 1). 

**Figure 2:**
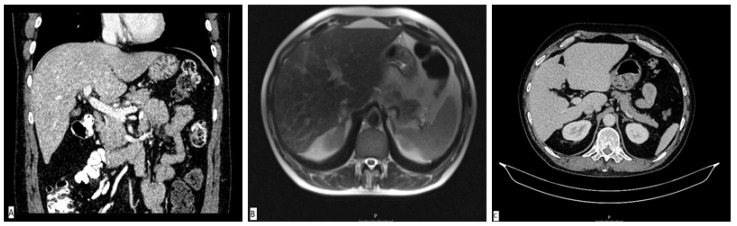
Radiological imaging of the liver parenchyma. MSCT scan of liver performed in March 2017 showing normal liver parenchyma (**A**). MR scan of liver performed in October 2017 showing a rough structure of the right liver lobe with initial retraction of the liver parenchyma, suggesting initial cirrhotic changes (**B**). Liver CT scan from March 2019 demonstrating liver cirrhosis (**C**).

A diagnostic panel excluded acute viral hepatitis as well as metabolic diseases, and the patient denied alcohol consumption or treatment with any concomitant drugs or supplements. An immunology panel revealed positive antinuclear (ANA), anti-mitochondrial (AMA) and AMA-M2 antibodies with increased immunoglobulin G (IgG) levels. Other autoimmune antibodies were negative. Liver sonography and an abdominal MSCT scan were ordinary (Figure 2A). He was treated with corticosteroid therapy (prednisone 1 mg/kg/day) and ursodeoxycholic acid (UDCA, 13 mg/kg/day), which led to a significant decrease in laboratory parameters. The calculated Roussel Uclaf Causality Assessment Method (RUCAM) score was 5, indicating the possible causality of the aforementioned supplement. 

**Figure 3:**
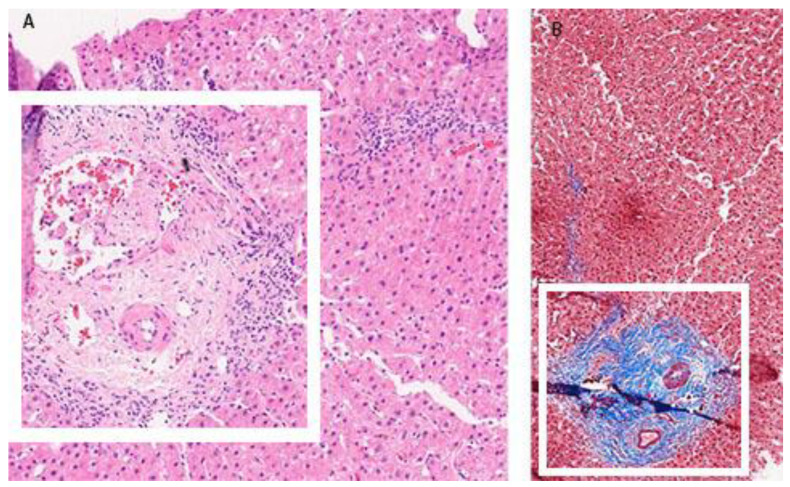
Pathohistological analysis of the liver biopsy specimen (December 2017). Hematoxylin and eosin staining: dilated portal space with a medium abundant mononuclear infiltrate (white frame) (**A**). Trichrome staining: blue staining of the fibrotic dilated portal space (white frame) (**B**).

The patient was perceived as having hepatocellular DILI and further treated as an out-hospital patient with tapering doses of corticosteroid therapy. In June 2017, three months after admission, corticosteroid therapy was withdrawn due to the complete normalization of laboratory parameters. In September 2017, an increase in aminotransferase levels was detected and the corticosteroid therapy was initiated again. In October 2017, liver MR was performed and described a rough structure of the right liver lobe, more pronounced in the peripheral zones, where coarser thickened septa with initial retraction of the liver parenchyma were observed, suggesting initial cirrhotic changes (Figure 2B). In December 2017, he was admitted again due to a significant increase in aminotransferase and bilirubin levels. A biopsy of the right liver lobe was performed, showing abundant T lymphocyte and plasma cell infiltration with numerous areas of piecemeal necrosis, without cholestasis. This finding corresponded with chronic active hepatitis, morphologically dominantly autoimmune hepatitis of medium activity (modified hepatitis activity index (mHAI) according to Scheuer 8–9/18) with liver fibrosis (stage 2/6) (Figure 3). After increasing the corticosteroid dose, a decrease in laboratory parameters was detected. He was further treated as an ambulatory patient with tapering corticosteroid doses and UDCA. The patient remained under biochemical control under low-dose corticosteroid therapy (prednisone 5–10 mg/day), with no further relapses. 

In March 2019, two years after the initial admission, a control abdominal MSCT scan was performed and showed an atrophic right liver lobe with irregular contours and a hypertrophic left lobe, indicating the presence of liver cirrhosis (Figure 2C). Since the left liver lobe seemed spared of fibrotic changes, it was biopsied and revealed insignificant inflammatory infiltrate without piecemeal necrosis, cholestasis or advanced fibrosis. 

In 2023, multiparametric ultrasound with elastography was performed and confirmed advanced liver fibrosis, with laboratory parameters indicating controlled inflammatory activity (normal bilirubin, aminotransferase and IgG levels). The immunology panel remained the same as in 2017. 

## 2. Discussion

DIAIH is a rare DILI phenotype, hardly distinguishable from classic idiopathic AIH, usually coupled with the antibiotic therapy (minocycline and nitrofurantoin), but also with other classes of drugs such as diclofenac, indomethacin, halothane, infliximab, methyldopa, hydralazine and statins [1,5]. Our patient was taking a muscle-building dietary supplement, and it is well known that their use may lead to hepatotoxic liver injury [6,7]. Regarding the pathophysiological pathway that underlies the clinical manifestation of DILI, Sebode et al. have discussed several points, including genetic background, antigen presentation, pro-inflammatory immune reactions, impaired drug metabolism and impaired regulatory mechanisms [7]. It is assumed that not a single defect, but their combination, may lead to an improper immunologic response. The immune reaction in DILI may be directed against the drug, its metabolites or the hapten, while in AIH, it is directed against the self-protein. Apart from the different antigen, the other distinction is the duration of antigen exposure. Additionally, antigen presentation may not be the only trigger for the inflammatory process, but perhaps also involves the second hit, usually in the form of viral infection, diet alterations, alcohol, trauma or others [7].

When trying to distinguish between idiopathic AIH and DIAIH, clinicians use a wide armamentarium of methods, ranging from a comprehensive patient history to genetic testing and liver biopsy. 

Regarding the clinical presentation, DIAIH tends to show a more severe clinical picture with jaundice, resulting in a higher rate of hospitalization requirement [8]. Patients usually present with nonspecific symptoms, including anorexia, fever, nausea, abdominal discomfort and malaise, sometimes followed by manifestations of hypersensitivity reactions [9]. They may also have a history of other autoimmune diseases or allergies and clinical symptoms depending on affection of other organs, such as the kidneys, lungs, skin, joints or gastrointestinal tract. Both disorders are accompanied by an increase in aminotransferase and bilirubin levels, without remarkable cholestasis. According to serology markers, 96% of patients with the DIAIH have positive autoantibodies characteristic of AIH (ANA, anti-smooth muscle antibody (ASMA), anti-liver-kidney microsomal antibody (anti-LKM)), and 90% of patients have elevated IgG levels. However, we must keep in mind the additional confusing factor of high antibody prevalence among asymptomatic individuals in the general population [1]. Additionally, carriers of HLA alleles DRB1*03:01/*04:01 have a higher risk of idiopathic AIH, while HLA DRB1*15:01 is known as the DILI-risk allele [1].

According to the Spanish DILI Registry, established in 1994, there were 26 DIAIH cases up to 2018, and the diagnosis was based on a convincing temporal relationship between the drug intake and the liver injury, no prior evidence of AIH, and the fulfilment of the simplified AIH criteria. When comparing features of patients with DIAIH with a complete DILI cohort, they revealed a higher predominance of female patients and a higher percentage of hepatocellular injury, as well as longer treatment requirement, in the DIAIH group [2].

According to the EASL guidelines, the performance of a liver biopsy in patients with suspected DILI is not obligatory, and may indeed be performed when the serological testing raises the possibility of AIH, but with a low level of evidence (Grade C, Level 4 studies) [1]. At the time of the initial patient presentation, we did not decide to perform a liver biopsy since we considered the diagnosis of DILI evident. Only after the exclusion of immunosuppressive therapy, when a relapse occurred, did we decide to perform it due to high suspicion of AIH.

Regarding histology patterns, DIAIH also presents with lymphocyte, eosinophilic and plasma cell infiltrates in portal and periportal spaces and interface hepatitis. According to Febres-Aldana et al., necroinflammatory and regenerative changes, as well as the portal and lobular densities of neutrophils and eosinophils, do not differ between the groups (*p* ≥ 0.05), but patients with idiopathic AIH more often show signs of collagen deposition and fibrosis (*p* < 0.05) [10]. Therefore, advanced stages of fibrosis support the diagnosis of AIH over DIAIH. Additionally, portal infiltrates in DIAIH are predominantly made of cytotoxic (CD8+) T cells, and of mature B cells (CD20+) in idiopathic AIH [1]. Still, there is no specific histopathologic sign that is pivotal for diagnosing DIAIH over AIH. Suzuki et al. compared pathohistological diagnosis among experts in the field, and found a unanimous agreement in only 46% of cases [11]. They proposed a model that included the number of plasma cells in portal spaces, portal inflammation, the number of eosinophils and lymphocytes in acinar spaces, the formation of rosettes and canalicular cholestasis. When testing the accuracy of the model, they found an AUROC of 0.90 in predicting DILI over AIH [11].

The most important distinguishment is provided at the end of the road, after the withdrawal of immunosuppression following the accomplishment of remission. Namely, DIAIH does not relapse over a long-term follow-up, while patients with idiopathic AIH relapse in 63% of cases in the first year after therapy cessation [10,12,13,14]. However, it has been reported that 10–18% of patients may develop chronic DILI during long-term follow-up [15]. He T. et al. analyzed data collected from 57 patients with recurrent DILI and found shortened latency periods and a higher incidence of chronic DILI following the recurrent episode (63.16% vs. 43.86%). Interestingly, 15 patients (26%) developed AIH during the second episode [15]. According to the USA-DILIN, chronic DILI is diagnosed when the liver disease persists 6 months after DILI onset [3,8]. In 2011, an international expert working group defined persistent DILI as abnormal liver biochemistry lasting more than 3 or 6 months for hepatocellular or cholestatic injury, respectively. They defined chronic DILI as liver injury lasting more than 12 months [16]. Similarly, in 2019. EASL defined chronic DILI as biochemical or imaging evidence of liver disease persisting one year after acute DILI onset [1]. Several forms of chronic DILI are described in the literature and include autoimmune-like DILI, vanishing bile duct syndrome, drug-induced steatohepatitis, secondary sclerosing cholangitis, sinusoidal obstruction syndrome, the development of fibrosis and, lastly, even liver cirrhosis with portal hypertension [5,12]. We emphasize that severe liver fibrosis following DILI occurs rarely, and is described only in isolated case reports [8]. 

Clinical presentations of DILI that mimic autoimmune liver disorders may lead to long-term unnecessary treatment with immunosuppressants. Therefore, in uncertain clinical scenarios, the best option would be gradual therapy cessation accompanied by regular patient monitoring. Regarding treatment with UDCA, although it is not common practice to administer it in patients with hepatocellular liver injury, we have witnessed its ability to lower bilirubin levels and to enhance liver regeneration. Studies have shown that apart from reducing the intestinal absorption of endogenous bile acids, UDCA has a cytoprotective, antiapoptotic and immunomodulator properties [17,18]. In addition to the treatment of cholestatic diseases, it has also been efficiently used in other indications-, e.g., neonatal hyperbilirubinemia and in individuals who have undergone liver donor hepatectomy [18,19]. The prognosis of patients with DILI will, among other factors, depend on the pathophysiologic mechanism that occurred after the drug exposure—whether it was a transitory liver injury or a trigger for the development of autoimmune disorder. Sebode M. et al. described several clinical scenarios that include both DILI and AIH: DILI development on top of AIH, drug-induced AIH, a second episode of DILI mimicking the relapse of AIH, chronic DILI mimicking AIH, and DILI with AIH characteristics [7]. When evaluating these possibilities, it is important to keep in mind that an individual may have two diseases simultaneously, such as DILI occurring in a patient who has unrecognized AIH. In doubtful cases, regular follow-ups of laboratory tests and a noninvasive assessment of liver fibrosis are mandatory.

Kumagai J et al. also described a case of AIH potentially triggered by two medications in an elderly patient. Treatment with low-dose corticosteroids led to complete remission, and the authors also left the question open regarding the final diagnosis [20]. 

Acute onset, the absence of advanced liver fibrosis and no relapse after corticosteroid withdrawal are the main features distinguishing DIAIH from idiopathic AIH. However, the diagnostic path of the presented patient seems to wander between the two disorders and leaves us in doubt, even years after the initial admission. Due to fulminant clinical onset with jaundice and according to the RUCAM-determined possible causality of the dietary supplement, he was initially perceived as having DILI, but the unexpected relapse after corticosteroid withdrawal and further development of liver cirrhosis eventually led to a diagnosis of idiopathic AIH. Could it be that our patient developed chronic DIAIH rather than classical AIH? Nevertheless, such a rapid development of liver fibrosis occurring in a 7-month interval seems too prompt. Did the patient already have existing liver fibrosis due to formerly unrecognized AIH and the drug acted as a trigger for severe necroinflammation and stimulus for the development of more advanced fibrosis? Regarding liver pathohistology, we have acquired knowledge of its insufficiency in making a definite distinction between the disorders in question.

## 3. Conclusions

Certain disorders have a typical clinical presentation and thereby a well beaten path regarding their diagnosis and treatment, which can often be simplified to plain algorithms. However, in our daily practice, we are faced with clinical scenarios where nothing goes by the book. These conditions are often among the spectrum of autoimmune diseases and require a significant amount of knowledge, experience and patience. Finally, certain medical cases never obtain a specific diagnosis. We must therefore keep in mind that regular patient monitoring and clinical open-mindedness with the adjustment of therapeutic approaches according to the disease course are more important than strict labeling of the disease. As Lewis JH charmingly pointed out, not all that glitters is necessarily the gold standard, and similarly, not all cases in clinical medicine are strictly categorizable into a certain box, leaving some questions open for good [21].

## 4. Future Directions

Future directions in this field include finding biomarkers and pathohistological indicators that could differentiate DILI from AIH, as well as conducting population-based cohort studies on the risk of supplementary dietary products. Scientists should focus on finding specific treatments that could reduce liver toxicity, and evaluate their efficacy in randomized controlled trials. According to the ClinicalTrials.gov online system, there is an ongoing retrospective, multi-center, non-interventional cohort study in China, with the intention of exploring whether artificial intelligence can discriminate between DILI and AIH (study ID:NCT05532345) [22].

## Data Availability

Data are available upon request.
